# Systematic evaluation of the meat qualities of free-range chicken (*Xuan-Zhou*) under different ages explored the optimal slaughter age

**DOI:** 10.1016/j.psj.2024.104019

**Published:** 2024-06-22

**Authors:** Guang Chen, Xun-yan Ling, Ming-shu Xie, Yang-fan Xiong, Ting-ting Li, Ying Wang, Cong-lin Shui, Chao-mu Li, Bao-cai Xu, Fei Ma

**Affiliations:** ⁎ School of Food and Biological Engineering, Hefei University of Technology, Hefei 230009, Anhui Province, China; † South Anhui Distinctive Agricultural Product Processing Technology Research and Application Center, Xuanzhou District Bureau of Agriculture and Rural Affairs, Xuancheng 242000, Anhui Province, China; ‡ Anhui Muzi Agriculture and Animal Husbandry Development Co., Ltd., Xuancheng 242000, Anhui Province, China

**Keywords:** Chicken, age, quality, analysis

## Abstract

Meat qualities of free-range chicken (*Xuan-Zhou*) (**XZ-FRC**) are closely associated with slaughter age and directly influence the economic benefits of supplier and consumer's preference. Understanding of the relationship between meat qualities and ages will be of prime important to explore a better slaughter age of XZ-FRC. In this study, the quality traits of breast and thigh muscles from XZ-FRCs at 9 to 14 wk were analyzed to establish a relatively reliable method for selecting a better slaughter age. The results showed that the effects of slaughter ages on color (CIE L*, a* and b* values), shear force, centrifugal loss, and flavor of XZ-FRCs were significant (*P* < 0.05). There were greater differences in meat qualities, whatever breast or thigh muscles, between same or different ages. Eleven feature indexes used for colligation evaluation of slaughter age were selected by combining the quality characteristics and data analysis. The score of colligation evaluation for XZ-FRCs at 12 wk was higher than that at 9 and 14 wk, suggesting that the 12 wk was an optimal slaughter age. This work would provide a reference method that helps the producers of livestock and poultry to select a better slaughter age.

## INTRODUCTION

Chicken meat, particularly breast and thigh muscles, is one of the most preferred choices by customers due to its rich nutrition composition, good taste, low price, and no religious restrictions (Kim et al., 2020; Li et al., 2022; Talukder et al., 2023). Free-range chicken (*Xuan-Zhou*) (**XZ-FRC**) according to the local standard protocol is defined as chicken that are adopted to the excellent conditions including a large-scale site located in forested mountainous area and a scientific feeding method. The free-range chicken is mostly preferred among consumers because of its health benefits such as animal welfare and meat traits as compared to conventional broilers, especially cage broilers (Jin et al., 2019; De Oliveira et al., 2023). At present, the XZ-FRCs have been farmed on a large-scale in Xuancheng city, south China's Anhui Province, and they were slaughtered over 20 million birds per year. Furthermore, in the global market, the overall consumption of free-range chicken meat also continues to increase (Cygan-Szczegielniak and Bogucka, 2021; Pinto da Rosa et al., 2021; Gál et al., 2022).

With the development of economy and society, the customers are paying more attention to high-quality chicken meat. Slaughter age is not only an important factor in affecting the chemical composition and quality of chicken meat (Li et al., 2019; Wang et al., 2020; Li et al., 2022a), but also closely related with economic benefits of commercial processors. For the moment, when a supplier needs to define a slaughter age of chickens, the quality traits after slaughter should be considered as the most critical indexes. However, the slaughter age of XZ-FRCs is selected as 14 wk entirely on the basis of the practical experience and feed conversion ratio. Thereby, it is particularly important to explore an optimal slaughter end point of the XZ-FRCs via evaluating their meat qualities. In addition, the decision of the optimal slaughter age will be also beneficial to manage some costs like feeding, labor, site, *etc*. It will be very exciting if the slaughter age of XZ-FRCs is less than 14 wk. Thus, this work will provide a theoretical basis for chicken producers to hold the future market.

Meat qualities are complex in nature and are commonly determined by a number of factors such as tenderness, color, centrifugal loss, and flavor (Cao et al., 2022). In fact, several of these factors like color, tenderness, *etc*. have been used to select the slaughter age of chicken based on sensory evaluation. It will be hard to make a scientific decision due to lack of systematic evaluation of meat qualities. To our knowledge, some producers decide the slaughter age of XZ-RFCs just rely on meat color of carcasses. The decision for the slaughter age is generally considered unreliable. Furthermore, there are significant difference in meat quality traits between chicken breast and thigh from the same breed, gender and age because of different fiber types, physicochemical compositions, and so on (Faria et al., 2022). It will be necessary for systematic evaluation of meat quality to use both chicken breast and thigh muscles as the research object. However, so far, no literature has been reported involving the selection of slaughter age of XZ-FRCs based on the systematic assessment of their quality traits.

The object of this study was to provide a scientific basis for selecting the slaughter age of XZ-FRCs by systematically analyzing the quality traits of both breast and thigh muscles. The main objects of this work were to 1) study the quality traits of breast and thigh muscles of XZ-FRCs including color, flavor, tenderness, and centrifugal loss at different slaughter ages; 2) explore a better slaughter age of XZ-FRCs based on colligation evaluation of meat qualities.

## MATERIALS AND METHODS

### Sample Preparation

A total of 44 XZ-FRCs for this work were provided by Anhui Muzi Agriculture and Animal Husbandry Development Co., Ltd., Chengxuan, China (118°28–119°04′E longitude, 30°34-30°19′N Latitude). All birds were male and reared in a forested mountainous with a radius of 2 km that was fenced with 2 m high plastic net. The birds had free daytime (from 06:00 to 18:00) but were confined to an indoor floor poultry house (5 birds/m^2^) at night. An appropriate feeding schedule was implemented to all birds according to the company's management procedures and the nutrient levels of basal diet were listed in Table 1.

**Table 1:** Components and nutrient levels of the basic diet for XZ-FRCs.#

Items	Content
**Ingredients**	
Corn	56.37
Green chaff	8.00
Soybean meal	13.73
Flour	4.00
Corn gluten meal	6.00
Rice DDGS	6.00
Oil	1.00
Bentonite	1.00
Stone powder	1.30
Premix (1%)^1^	2.60
Total	100.00
**Calculated nutrient levels (%)**	
Crude protein	19.00
Metabolizable energy (MJ·kg^−1^)	3.05
Calcium	0.80
Phosphorus	0.60
Available phosphorus	0.38
Lysine	0.90
Methionine and cysteine	0.70
Threonine	0.60
Arginine	1.05
Tyrosine	0.16

# Premix provided per kg of diet: vitamin A, 40,000,000 IU; vitamin D3, 12,500,000 IU; vitamin E, 100,000 IU; vitamin K3, 8,000 mg; vitamin B1, 6,000 mg; vitamin B2, 25,000 mg; vitamin B6, 15,000 mg; vitamin B12, 75.0 mg; D-Biotin, 500.0 mg; D-Pantothenic acid, 50,000 mg; folic acid, 4,500 mg; nicotinamide, 160,000 mg; moisture content was less than 10%.

During the experiment, the indoor and outdoor environmental conditions were and similar and basically stable. The average temperature and relative humidity of them were 22 ± 2°C and 70 ± 5%, respectively. Before they were placed into the free-range area, also known as chick period, the temperature for the age of 1 to 3 d, 4 to 7 d, 2, 3, 4, and 5 to 8 wk was 35 to 36°C, 33 to 34°C, 31 to 32°C, 28 to 30°C, 25 to 27°C and 18 to 22°C, respectively. Moreover, the relative humidity for the age of 1 to 3 d, 4 to 7 d and 2 to 8 wk was 60 to 70%, 50 to 60%, and 65 to 70%, respectively.

In this work, the number of birds at 9, 12, and 14 wk of age used for slaughter was 21, 16, and 7, respectively. The bird of different age groups was separately raised in corresponding free-range area. The birds for experiment (44 samples) were captured randomly from the poultry houses at night (from 20:00 to 22:00) and followed by slaughtered according to the operating procedures of poultry slaughtering-chicken (GB/T 19478-2018). The electrical water-bath stunning was firstly performed for 10 s at 60 to 80 v. Then, the birds were killed, exsanguinated (3–5 min), scalded (58–62°C), removing the feathers and internal organs, cleaned, *etc.*, in turn. All research protocols were approved by the Hefei University of Technology Ethical Committee on Animal Research (Permit numbers: HFUT20220406001 and HFUT20240104001). The average weight of XZ-FRCs at 9, 12, and 14 wk was 0.96±0.08, 1.34±0.13 and 1.47±0.15 kg, respectively.

The breast and thigh muscles of carcasses were collected and their visible fat, connective tissue and bone were immediately removed. The obtained breast muscles from each carcass were randomly divide into 2 groups, and followed by packaged with vacuum soft packing and stored at -20°C. After thawing for about 24 hours at 4°C, one group was directly used to determine the color, tenderness, and centrifugal loss and the other group was ground with a meat grinder (S1-M81(D), Joyoung Co., Ltd., China) before detecting the free amino acids, flavoring nucleotides, and volatile flavor compounds. The similar operations were also performed in the obtained thigh muscles.

### Determination of Quality Traits

*Color.* The color of breast and thigh muscles was evaluated using a colorimeter (ZE-7700, Nippon Denshoku, Japan) according to a modified procedure originally reported by Deng et al. (2022). The colorimeter had an illuminant D65, a 10° standard observer position, and a 4.0-mm diameter aperture and was calibrated with a white plate and a dark plate before analysis. The CIE L*, a* and b* values of each sample were measured in triplicate from different sites. Finally, the color values of each sample were expressed by the average of these readings.

*Tenderness.* The tenderness of breast and thigh muscles was expressed by shear force that was conducted using the Meullenet-Owens razor shear (**MORS**) method, as described by Polášek et al. (2021) with slight modification. A texture analyzer (TA-XT plusC, Stable Micro System Co., UK) equipped with a 5-kg load cell was applied to collect MORS data. All muscles were firstly cut and shaped into cuboids with dimensions of approximately 1.5 × 1.5 × 0.5 cm (length × width × height for each cuboid). Each cuboid was cut 3 time vertical to the muscle fiber direction using a shear blade (0.5-mm thick, 8.9-mm wide, and 30 mm in height) to acquire an average value for shear force. The blade penetrated 25 mm in each cuboid at a trigger type button with a 2.0 mm/s pretest speed, a 5.0 mm/s test speed, a 5.0 mm/s post-test speed, and a 5 g trigger force.

Centrifugal loss. Centrifugal loss of breast and thigh muscles was measured using a centrifuge (VELOCITY 14R, Dynamica Pty. Ltd., Australia) according to Ma et al. (2013) and Zhu et al. (2019) with slightly modification. A muscle sample of about 5 g was firstly collected from a breast or thigh. The weight of the muscle sample was recorded as M_1_ after wiping the surface water with absorbent paper. The obtained sample was then wrapped in filter paper and placed in a 50 mL centrifuge tube and centrifuged at 990 × *g* for 10 min at 4°C. Finally, the centrifuged sample was weight and recorded as M_2_. The centrifugal loss was defined according to the following equation.

(1) $$Centrifugal\;loss=\frac{\left(M_{1}-M_{2}\right)}{M_{1}}\times 100\%$$

Free amino acid. The free amino acids of breast and thigh muscles were extracted and detected using an automatic amino acid analyzer (S-433D, Sykam, Germany) referring to Qi et al. (2021) and Li et al. (2022b) A muscle sample of 3 g was freeze dried in a vacuum freeze dryer (FD-1A-50, Beijing Boyikang Lab Instrument Co., Ltd., China) for 24 h and then calculated its moisture content (%). The dried sample of 0.1 g was placed in a 10 mL centrifugal tube and 4 mL of 4% (*w*/*v*) 5-sulfosalicylic acid was added. The mixture was homogenized for 30 min using an ultrasonic instrument (KS-7200XDS, Kunshan Jielimei Ultrasonic Instrument Co., Ltd., China) combined with manual shake, and then was centrifuged for 30 min at 13,800 × *g* (4°C) using the VELOCITY 14R freezing centrifuge. The clear supernatant was filtered through a 0.22 μm water-phase filter membrane. Afterwards, the free amino acids in filtrate were analyzed by comparing their retention times and peak areas with the corresponding standard amino acids. Finally, the content of free amino acids in the muscle sample was calculated as follow:

(2) Freeaminoacid(mg/100g)=F(mg/100g)×[1−W(%)]

where *F* and *W* were the content of free amino acids in dried muscle sample and the moisture content of muscle sample calculated by above freeze dried.

*Volatile flavor compound.* The volatile flavor compounds of breast and thigh muscles from XZ-FRCs were determined using a modified method reported by Shen et al. (2023). Five grams of muscle sample was placed into a 20 mL sealed headspace vial and stored at 4°C for further measurement. A 50/30µm DVB/CAR/PDMS extraction fiber head (57330-U, Supelco, US) was inserted into the sealed headspace vial after incubating for 40 min at 60°C in a constant temperature water bath. The adsorption process of flavor compounds lasted 20 min. The extraction head was then inserted into the injection port of a GC-MS analyzer (8890-5977B, Agilent Technologies Inc.) and desorbed at 250°C for 5 min. The carrier gas was ultra-pure helium (> 99.9%) and its flow rate was 1 mL/min. An HP-5MS capillary column (30 m × 0.25 mm × 0.25 µm) was used to separate the flavor compounds. The heating procedure was as follows: heated up to 40°C and kept for 2 min, increased to 120°C at a rate of 5°C/min and kept for 5 min, then increased to 200°C at a rate of 5°C/min and maintained for 2 min, and finally heated to 250°C with a rate of 8°C/min and kept for 8 min. The mass spectrometry conditions were as follows: the temperature of chromatography-mass spectrometry interface, ion source and quadruple rod were 280°C, 230°C, and 150°C, respectively; the ionization mode was electron impact (EI); the electron energy and mass scan range were 70 eV 30-550 (*m/z*), respectively. Finally, the volatile compounds were discovered and identified by comparing their mass spectra with those in the National Institute of Standards and Technology (**NIST**) 20 library. The content of all volatile compounds was calculated by comparing the peak area of each compound with that of the corresponding internal standard.

*Flavoring nucleotide.* In this work, 5՛-nucleotides as representative flavoring nucleotides in breast and thigh muscles from XZ-FRCs were determined on the basis of the methods reported by Qi et al. (2021) and Yin et al. (2022) with slightly modification. Five grams of muscle sample was mixed with 20 mL of 10% perchloric acid (*w/w*) and then homogenized for 5 min, followed by ultrasonication for 10 min in an iced-water bath. Afterwards, the mixture was centrifuged at 10,766 × *g* for 10 min at 4°C and the obtained precipitate was subjected to a second extraction. The supernatant was combined from two extracts and adjusted immediately to pH 5.70., followed by standing for 1 h at 4°C. After filtration with a 0.22 μm microporous membrane, the clear supernatant was analyzed by a HPLC system (LC-2030C 3D plus, Shimadzu, Japan) equipped with a SB-C18 chromatographic column (5 μm, 4.6 × 250 mm) and a diode array detector. The column temperature and UV detector wavelength were 30°C and 254 nm, respectively. The mobile phase used were (A) potassium phosphate buffer (0.015 M KH_2_PO_4_, 0.015 M K_2_HPO_4_, 1 g C_16_H_37_NO_4_S, pH 5.70) and (B) methanol. Ultrasonic degassing of the two mobile phases was performed for 30 min before elution. The flow rate was 1.0 mL/min and the elution mode was isocratic elution with mixture of 96.5% A and 3.5% B. The content of all flavoring nucleotides was calculated by comparing the peak area of each nucleotide with that of the corresponding internal standard. The *R*^2^ of the flavor nucleotides standard curves all reached 0.9999.

### Data Analysis

*Statistical analysis.* All experimental results of meat quality were analyzed using SPSS Statistic 22.0 (software v.10.0.2, Minnesota) and the means between the research groups were compared by one-way analysis of variance with Duncan's test. The significance of difference was set as *P*<0.05.

*Colligation evaluation of slaughter age.* To get a better slaughter age of XZ-FRCs within 9 to 14 wk, the feature indexes were firstly selected from color (L*, a* and b*), tenderness,centrifugal loss, free amino acid, volatile flavor compound and flavoring nucleotide based on their characteristics combined with data analysis such as meta-analysis, Pearson correlation analysis and threshold analysis. The selected feature indexes were used to establish a relatively reliable evaluation method based on the calculation of colligation score, which was defined by the following equation (3). Each value of the selected features was dealt with normalization before computing the colligation score. The weight coefficient of all feature indexes was -1 or 1 based on their function for meat qualities. However, meat color was a non-standard sensory quality for XZ-FRCs with 9 to 14 wk because of lacking sensory thresholds of CIE L*, a* and b* values. Therefore, it was hard to define the weight coefficients for CIE L*, a* and b* values. To further evaluate the effect of slaughter age on color and colligation quality, the CIE L*, a* and b* values were also analyzed by meta-analysis and Pearson correlation analysis. The weight coefficient of shear force (tenderness) and centrifugal loss, as two feature indexes, was set to -1. As part of the feature indexes, some flavor compounds were selected from free amino acid, volatile flavor compound and flavoring nucleotide when their relative contents were more than corresponding sensory thresholds.

(3) $$\text{Colligation}\;\text{score}=\sum_{i}^{n}m_{i}X_{i}$$

where *m* is the weight coefficient; X is the normalized value of feature index; and n is the number of feature index.

## RESULTS AND DISCUSSION

### Color

**Figure 1:**
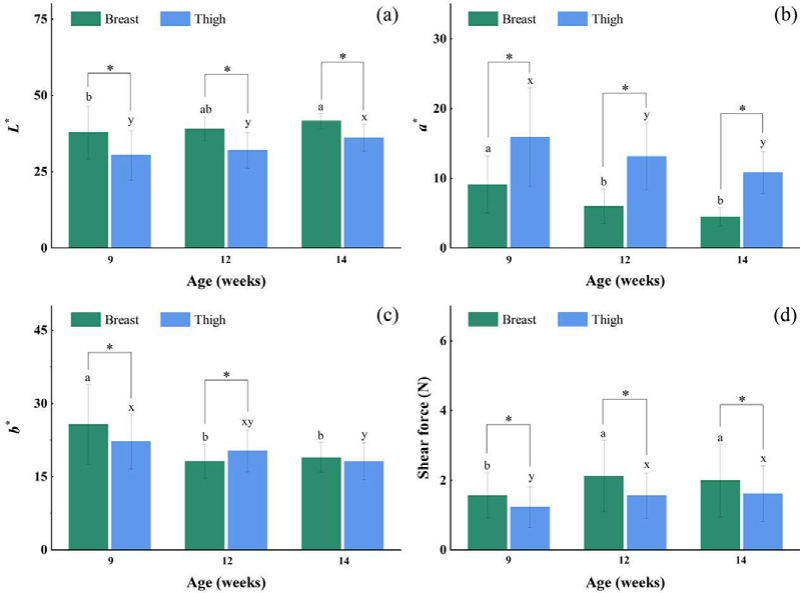
The average values of L* (a), a* (b), b* (c) and shear force (d) of breast and thigh muscles from XZ-FRCs at different ages (9–14 wk). Vertical bars represent standard deviations from at least seven samples, and each of them was repeated three times. * means in the same ages are significantly different (*P* < 0.05). a-c and x-y means in the different muscles with different letters are significantly different (*P*<0.05).

Color is a key indicator for assessing the meat quality of XZ-FRCs that directly affects consumer's purchasing intentions. The CIE L*, a* and b* values of XZ-FRCs at different ages were shown in Figure 1. With the increase of age, the L* values of breast and thigh muscles were gradually increasing, while the a* and b* values of them showed decreasing trends. The L* values of breast and thigh muscles at 14 wk were obviously higher than those at 9 wk (*P* < 0.05), while the variations of b* values were just the opposite. The breast and thigh muscles from XZ-FRCs at 9 wk had significantly higher a* values than those at 12 to 14 wk (*P* < 0.05). The differences in the L*, a* and b* values between breast and thigh muscles at different ages (9–14 wk) were significant (*P* < 0.05) except the b* value at 14 wk. The a* value of thigh muscles at 9 to 14 wk was obviously higher than that of breast muscles (*P* < 0.05). Previous literature reported that the L* values of chicken muscles presented an upward trend with age, and the a* and b* values of chicken muscles decreased with age (Katemala et al., 2021). The reason of these similar results was possibly due to the changes in myoglobin content during growing (Katemala et al., 2021). However, a completely opposite result for the a* values was reported in a literature (Deng et al., 2022), which agreed that higher a* values of Tuer chickens (Deng et al., 2022) might be caused by higher levels of myoglobin with increasing age. This contradictory phenomenon may be due to the influence of free-range chicken breeds, rearing patterns and environment.

### Tenderness

Tenderness is one of the important elements of eating quality and generally evaluated by Warner-Bratzler shear force (Mennecke et al., 2007; Shi et al., 2021). As shown in Figure 1, the tenderness of breast muscles was significantly lower than that of thigh muscles for the XZ-FRCs at each age (9–14 wk) (*P* < 0.05) except at 14 wk. A similar result involving the comparison of tenderness between the breast and thigh muscles was also reported by Deng et al. (2022). Compared with the 9 wk, the breast and thigh muscles of XZ-FRCs at 12 to 14 wk had obviously lower tenderness (higher shear force) (*P* < 0.05). Within the age range of 9 to 14 wk, the shear force of breast and thigh muscles was no longer increased significantly (*P* > 0.05) when the age of XZ-FRCs was more than 12 wk. In general, the tenderness of chicken muscles including breast and thigh presented a downward trend with age (López-Pedrouso et al., 2019), which was largely attributed to the changes of physicochemical properties like collagen content, connective tissue solubility, muscle fiber structure, and so on (Kong et al., 2008; Deng et al., 2022).

### Centrifugal Loss

**Figure 2:**
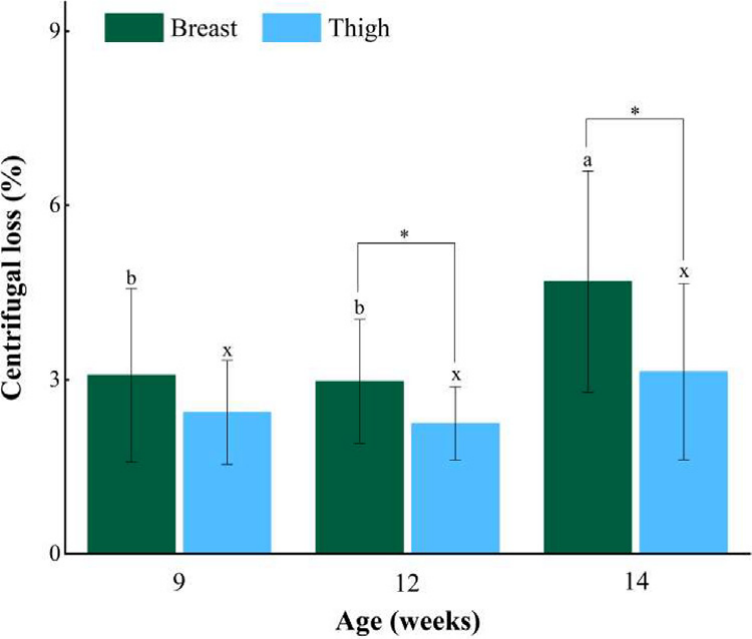
The average values of centrifugal loss of breast and thigh muscles from XZ-FRCs at different ages (9–14 wk). Vertical bars represent standard deviations from at least seven samples. * means in the same ages are significantly different (*P* < 0.05). a-b means in the different muscles with different letters are significantly different (*P* < 0.05).

Centrifugal loss is a very important element that is directly related to the changes of nutritional ingredients and processing qualities of chicken meat (Cao et al., 2022). As shown in Figure 2, the centrifugal loss of breast muscles was significantly higher than that of thigh muscles for XZ-FRCs at 12 to 14 wk (*P* < 0.05). The centrifugal loss of breast muscles at 14 wk was significantly higher than that of the muscles at 9 to 12 wk suggesting that the breast muscles from XZ-FRCs at 14 wk had a relatively weak water-holding capacity. Thus, more nutrients could be retained in the breast muscles at 9 to 12 wk compared with 14 wk. No significant difference in centrifugal loss was observed for thigh muscles from XZ-FRCs among the three ages (9, 12, and 14 wk) (*P* > 0.05) (Figure 2). In general, the water-holding capacity of meat mainly depended on the affinity between protein and water molecules and the spatial morphologies constructed by muscle fibers (Jung et al., 2015). With age increasing, the muscle fibers in chicken are thickening (Li et al., 2019), which might change the space to retain water within the muscle fibers in XZ-FRCs and further influenced the centrifugal loss of breast and thigh muscles. Katemala et al. (2021) reported that the centrifugal loss of breast muscles from Korat hybrid chickens decreased with increasing age (*P* < 0.05), which was opposite to the results of this work. However, for thigh muscles, whatever Korat hybrid chickens (Katemala et al., 2021) or XZ-FRCs, the change trends of centrifugal loss were similar. These interesting results will be worthy of further study.

### Free Amino Acids

The contents of sixteen flavor-presenting amino acids and their taste threshold were shown in Table 2. The contents of all fresh and sweet amino acids (except valine) in breast muscles were obviously lower than those in thigh muscles from XZ-FRCs at the same age (*P* < 0.05), while the results of bitter amino acids (except arginine) were just the opposite except at 14 wk. Total free amino acids for umami and sweet, whatever breast or thigh muscles, at 12 wk were higher than those at 14 wk. The contents of aspartic acid (**Asp**), threonine (**Thr**), valine (**Val**), and isoleucine (**Ile**) in breast muscles from XZ-FRCs at 12 wk were significantly higher than those at 14 wk (*P* < 0.05). However, no significant differences in umami and sweet amino acid contents were observed for thigh muscles among different ages (9–14 wk) except Thr (*P* > 0.05). Besides, the content of glycine (**Gly**) in breast muscles had an obvious decrease trend with increasing age (*P* < 0.05). For bitter amino acids, the contents of methionine (**Met**) and tyrosine (**Tyr**) in thigh muscles at 12 wk were significantly lower than those at 14 wk. By comparing analysis, the above results implied that the 14 wk as slaughter end point of the XZ-FRCs, to a large extent, might not obtain an optimal meat quality.

**Table 2:** The contents of free amino acids (mg/100g) in breast and thigh muscles from XZ-FRCs at different ages (9, 12, and 14 wk).#

Amino acids	Three ages for breast muscles			Three ages for thigh muscles			Threshold value
	9	12	14	9	12	14	
Umami free amino acids (UFAA, mg/100g)							
Aspartic acid (Asp)	6.62±1.53^by^	8.12±1.87^ay^	6.78±1.82^by^	25.60±10.76^ax^	27.31±10.64^ax^	21.75±7.62^ax^	100
Glutamic acid (Glu)	19.25±8.74^ay^	20.47±5.58^ay^	17.47±4.37^ay^	46.28±18.88^ax^	48.26±15.84^ax^	38.35±13.15^ax^	30
Total UFAA	25.54±8.72^ay^	28.08±6.57^ay^	23.28±6.80^ay^	71.87±28.01^ax^	75.57±25.02^ax^	60.10±20.52^ax^	/
Sweet free amino acids (SFAA, mg/100g)							
Threonine (Thr)	15.22±4.53^aby^	18.47±5.95^ay^	12.19±2.71^by^	58.24±20.47^ax^	61.08±13.75^ax^	42.27±8.07^bx^	260
Serine (Ser)	12.19±4.30^ay^	13.09±3.03^ay^	11.96±2.55^ay^	25.78±9.50^ax^	22.29±4.95^ax^	22.81±6.91^ax^	150
Lysine (Lys)	8.09±3.58^ay^	7.43±2.08^ay^	7.08±1.31^ay^	12.81±8.57^ax^	11.68±2.60^ax^	12.66±3.72^ax^	50
Glycine (Gly)	8.27±1.57^ay^	7.62±1.17^aby^	6.47±1.32^by^	14.76±3.67^ax^	14.96±2.39^ax^	14.95±4.39^ax^	130
Alanine (Ala)	14.65±3.97^ay^	12.90±4.00^ay^	15.11±4.74^ay^	29.85±7.67^ax^	25.18±5.10^ax^	31.63±9.03^ax^	60
Valine (Val)	3.63±1.17^abx^	4.57±1.22^ax^	3.40±0.55^bx^	3.31±1.15^ax^	3.84±0.78^ax^	3.61±0.95^ax^	40
Total SFAA	61.46±14.86^ay^	63.32±11.47^ay^	54.81±10.66^ay^	143.3±35.46^ax^	137.5±13.85^ax^	123.4±28.71^ax^	/
Bitter free amino acids (BFAA, mg/100g)							
Cystine (Cys)	ND	ND	ND	ND	ND	ND	/
Methionine (Met)	2.39±0.78^ax^	2.49±0.92^ax^	2.51±0.82^ax^	1.70±0.47^aby^	1.46±0.28^by^	2.09±0.81^ax^	30
Isoleucine (Ile)	2.71±0.92^bx^	3.64±1.31^ax^	2.51±0.59^bx^	2.13±0.73^ay^	2.66±0.75^ay^	2.19±0.62^ax^	90
Leucine (Leu)	5.18±1.80^ax^	5.84±1.93^ax^	5.08±1.46^ax^	4.12±1.16^ay^	4.35±0.98^ay^	4.25±1.13^ax^	190
Tyrosine (Tyr)	4.96±1.09^ax^	3.94±0.85^bx^	4.83±1.19^abx^	3.60±1.06^ay^	2.64±0.63^by^	3.48±1.12^ax^	91
Phenylalanine (Phe)	3.62±1.00^ax^	3.10±0.79^abx^	2.93±0.65^bx^	2.62±0.64^ay^	2.29±0.45^ay^	2.41±0.68^ax^	90
Histidine (His)	124.0±18.20^ax^	125.1±25.92^ax^	124.74±7.35^ax^	63.60±17.21^ay^	64.97±17.43^ay^	54.46±17.26^ay^	20
Arginine (Arg)	4.33±2.10^ay^	4.81±1.62^ay^	4.44±2.21^ax^	6.46±2.11^ax^	7.10±1.41^ax^	6.31±2.56^ax^	50
Total BFAA	146.5±22.09^ax^	148.7±28.06^ax^	146.4±12.69^ax^	83.89±19.66^ay^	85.48±18.45^ay^	75.19±22.45^ay^	/

# ND: Not detected; Each value is expressed as mean ± SD (n≥7). ^a-c^ Mean values in the different ages with different superscripts differ significantly (*P* < 0.05) for same muscle type; ^x-y^Mean values in the same age with different superscripts differ significantly (*P*<0.05) for different muscle type. UFAA, BFAA and SFAA represent umami free amino acids, bitter free amino acids and sweet free amino acids, respectively. The UFAA, BFAA and SFAA was defined based on the reports of Duan et al. (2023) and Yuan et al., 2021. Threshold values of taste were set according to the report of Yuan et al., 2021.

**Figure 3:**
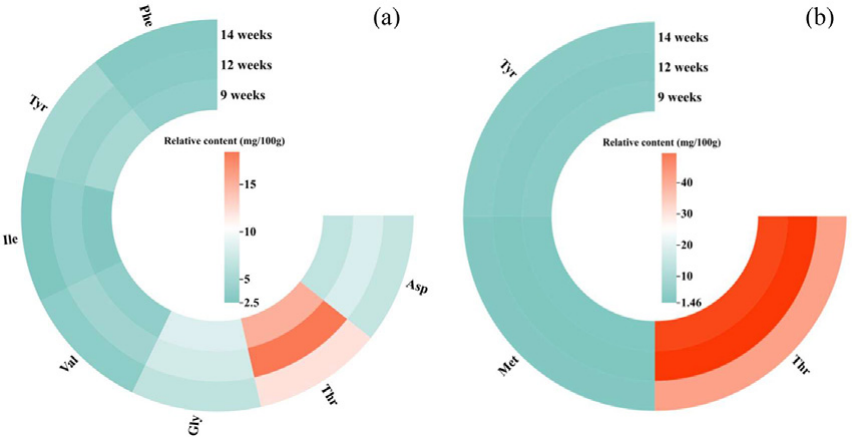
Heatmap of free amino acids in breast (A) and thigh (B) muscles with significant changes. Asp ‒ aspartic acid; Thr ‒ threonine; Gly ‒ glycine; Val ‒ valine; Ile ‒ isoleucine; Tyr ‒ tyrosine; Phe ‒ phenylalanine; Met ‒ methionine.

To easier display the contents of free amino acids that changed significantly with age, a heatmap was designed as shown in Figure 3. For breast muscles, 1 umami amino acid (**Asp**), 2 sweet amino acids (Gly and Val) and three bitter amino acids (Ile, Tyr, and phenylalanine) were significantly changed by slaughter age and their results were shown in Figure 3A. Only three amino acids, including sweet (Thr) and bitter (Tyr and Met), in thigh muscles changed significantly with different slaughter age (Figure 3B). Although the contents of glutamic acid (**Glu**) in breast muscles and histidine (**His**) in breast and thigh muscles were higher than the sensory thresholds that were defined by Yuan et al. (2021) and Qi et al. (2017), they were not significantly affected by slaughter age (*P* > 0.05). As the 2 amino acids (Glu and His) with higher contents (Table 2), they probably had interaction effects with other amino acids listed in Figure 3.

### Volatile Flavor Compounds

In this work, a total of 56 volatile compounds in breast and thigh muscles from XZ-FRCs at different ages were measured and their results were shown in Table 3. These volatile compounds mainly included 13 aldehydes, 8 alcohols, 7 ketones, 17 hydrocarbons, 2 esters, 1 ether, and 8 other flavor compounds (2-pentylfuran, 1,3-propanediamine, *etc*.). For sensory thresholds, thirty-six flavor compounds of them have specific reference values (Gemert, 2011; Fan et al., 2018; Shen et al., 2023) and while the other 20 compounds were not found in existing literatures. Under current information, there were only six compounds (hexanal, heptanal, octanal, nonanal, dodecanal and 1-octen-3-ol) in breast and thigh muscles that their concentrations in all or several experiment samples were more than the corresponding sensory thresholds, and the six compounds were used to the colligation evaluation of slaughter age (Table 3). An important reason, for the XZ-FRCs with different ages, was that the six compounds would be easier perceived by the human senses than the other 50 compounds.

**Table 3:** Volatile flavor components (ng/g) in breast and thigh muscles from XZ-FRCs at different ages (9–14 wk).#

RT	Compound	Three ages for breast muscles			Three ages for thigh muscles			ST
		9	12	14	9	12	14	
**Aldehydes**								
6.29	Pentanal	4.12±3.71^ax^	5.50±4.74^ax^	6.47±3.58^ax^	6.49±5.17^ax^	2.83±2.64^abx^	1.40±1.10^by^	12
8.87	Hexanal	60.9±51.9^ax^	144.2±145.8^ax^	52.7±36.2^ax^	63.7±68.6^ax^	47.20±58.61^ay^	16.16±15.21^ay^	5
10.92	2-Propenal	0.45±0.26^ax^	/	0.43±0.20^ax^	0.42±0.32^ax^	0.20±0.12^ax^	0.32±0.06^ax^	10.92
11.92	Heptanal	1.75±1.59^abx^	3.96±4.11^ax^	0.64±0.38^bx^	2.01±1.88^ax^	2.16±2.44^ax^	0.30±0.26^ay^	2.8
13.83	Benzaldehyde	1.80±1.13^ax^	1.56±0.83^ax^	0.82±0.70^ax^	1.67±0.68^ax^	0.71±0.40^by^	/	750.89
15.01	Octanal	1.44±0.85^ax^	3.70±4.88^ax^	0.50±0.03^ax^	2.27±1.32^ax^	1.82±2.08^abx^	0.37±0.19bx	0.59
16.67	(E)-2-Octenal	/	1.08±0.68^ax^	/	1.27±0.80^ax^	0.83±0.82^ax^	/	3
18.00	Nonanal	2.35±1.52^bx^	6.89±7.53^ax^	0.66±0.23^by^	4.38±4.01^ax^	3.66±3.26^ax^	1.93±1.73^ax^	1.1
21.44	Decanal	0.51±0.20^by^	2.64±1.10^ax^	/	1.14±0.47^ax^	1.31±0.65^ay^	/	3
29.52	Dodecanal	/	0.60±0.16^ax^	/	/	/	/	M_1_
32.58	Tridecanal	/	0.63±0.31^ax^	/	/	/	/	10
35.25	Tetradecanal	/	0.75±0.42^ax^	/	/	/	/	2.1
37.64	Pentadecanal	/	0.81±0.70^ax^	/	/	/	/	1000
**Alcohols**								
6.27	DS՛	9.44±7.22^bx^	9.96±7.95^bx^	25.1±22.1^ax^	11.1±8.06^bx^	9.49±4.33^bx^	22.34±12.53^ax^	/
7.97	1-Pentanol	3.21±2.33^ax^	4.51±4.18^ax^	3.57±0.68^ax^	5.82±4.17^ax^	4.51±4.07^ax^	1.43±0.08^ay^	150.2
8.76	1-Butanol	0.44±0.45^ax^	/	/	0.84±0.93^ax^	0.66±0.51^ax^	/	459.2
10.92	1-Hexanol	0.53±0.16^bx^	1.40±1.00^ax^	/	1.56±1.40^ax^	1.26±0.66^ax^	/	5.6
13.81	2,5-D-3-H	2.13±0.26^ax^	2.08±0.20^ax^	/	2.28±0.28^ax^	2.13±0.12^ax^	/	M_2_
14.28	1-Octen-3-ol	5.03±5.36^ax^	17.47±21.34^ax^	2.81±1.80^ax^	5.62±7.10^ax^	5.60±8.04^ay^	2.08±1.23^ay^	1.5
15.76	2-Ethylhexanol	24.55±17.93^abx^	38.32±30.85^ax^	11.0±4.62^bx^	14.9±14.5^by^	7.62±6.71^by^	34.31±23.58^ax^	M_3_
16.64	1,4-Butanediol	0.70±0.99^ax^	0.10±0.04^ax^	0.24±0.30^ax^	0.23±0.18^ax^	/	/	M_4_
**Ketones**								
3.64	Acetone	10.79±6.47^ax^	9.92±4.45^ax^	18.1±15.4^ax^	11.0±8.42^bx^	13.39±8.05^bx^	23.86±6.48^ax^	832
4.57	2-Butanone	2.13±1.38^ax^	0.68±0.39^ax^	2.70±0.81^ax^	1.73±1.23^ax^	0.91±0.67^ax^	1.66±0.69^ax^	M_5_
11.47	3-Heptanone	2.14±1.06^bx^	1.68±0.56^bx^	3.21±0.59^ax^	2.3±1.4^abx^	1.70±0.60^bx^	3.00±0.63^ax^	M_6_
14.09	4-Octanone	0.70±0.47^ax^	0.75±0.39^ax^	0.68±0.41^ax^	0.54±0.26^ax^	0.56±0.29^ax^	0.70±0.17^ax^	M_7_
14.20	2,4-D-3-H	2.00±1.32^ax^	1.51±0.72^ax^	3.60±1.16^ax^	1.99±1.47^bx^	1.60±0.89^bx^	3.88±0.30^ax^	/
14.21	6-M-5-H-2-O	0.62±0.37^ax^	0.32±0.13^ax^	/	0.75±0.29^ax^	0.67±0.29^ax^	/	68
35.26	3-Penten-2-one	0.19±0.15^bx^	0.08±0.04^bx^	0.43±0.20^ax^	0.17±0.09^ax^	/	/	1200
**Hydrocarbons**								
3.69	S-(-)-1,2-E	8.76±3.97^ax^	12.40±5.44^ax^	/	7.97±2.35^ax^	/	/	/
3.92	Methylene chloride	0.88±0.41^ax^	0.50±0.16^aby^	0.38±0.03^bx^	1.13±0.79^ax^	0.92±0.17^ax^	0.55±0.30^ax^	5600
4.56	Pentane	1.02±0.71^ax^	0.82±0.23^ax^	/	1.38±0.39^ax^	0.90±0.34^abx^	0.57±0.28^bx^	/
4.86	Trichloromethane	1.74±2.20^ax^	0.97±0.51^ax^	1.47±0.90^ax^	1.38±0.69^ax^	1.11±0.59^ax^	1.26±1.01^ax^	120
8.02	Toluene	3.93±2.76^ax^	4.19±3.27^ax^	1.36±0.36^ax^	4.38±2.75^ax^	4.38±2.57^ax^	1.47±0.84^ax^	527
9.22	Tetrachloroethylene	0.26±0.12^ax^	0.94±0.52^ax^	/	0.54±0.28^bx^	1.36±0.85^ax^	/	240
9.47	HC՛	15.02±6.44^ay^	12.25±6.48^ax^	13.6±7.16^ax^	21.8±9.42^bx^	13.08±4.86^bx^	55.84±105.4^ax^	/
10.80	Ethylbenzene	0.54±0.38^ax^	0.42±0.19^ax^	/	0.67±0.29^ax^	0.49±0.24^ax^	0.42±0.25^ax^	M_8_
11.01	m-Xylene	0.65±0.29^ay^	0.61±0.25^ax^	/	0.99±0.39^ax^	0.76±0.39^ax^	/	1000
11.70	1,3,5,7-C	1.35±0.89^ax^	0.88±0.31^ax^	/	2.33±1.06^ax^	1.30±0.66^ax^	/	/
14.76	OCS՛	83.88±76.53^ax^	61.95±46.40^ax^	78.1±52.3^ax^	96.7±51.4^ax^	56.58±25.79^bx^	77.70±47.04^abx^	/
15.87	D-Limonene	0.35±0.22^ax^	/	0.23±0.08^ax^	/	/	0.40±0.18^ax^	34
16.63	4,7-D	0.44±0.36^ax^	/	0.45±0.17^ax^	/	/	0.47±0.44^ax^	/
19.42	DCS՛	59.96±56.39^ax^	45.52±32.75^ax^	31.4±13.5^ax^	63.4±44.1^ax^	42.04±25.62^abx^	27.93±16.29^bx^	/
26.58	DC՛	13.26±12.18^ax^	10.35±6.71^ax^	4.90±1.56^ax^	12.5±8.17^ax^	10.84±6.58^ax^	3.37±1.14^by^	/
36.57	HC՛	0.93±0.68^ax^	0.90±0.46^ax^	/	0.64±0.21^ax^	0.79±0.48^ax^	/	/
40.13	OC՛	0.40±0.25^ax^	0.42±0.18^ax^	/	0.32±0.09^ax^	0.41±0.21^ax^	/	/
**Esters**								
4.83	Ethyl acetate	0.50±0.18^ax^	0.46±0.51^ax^	0.60±0.54^ax^	/	0.21±0.16^ax^	/	5
6.09	AAEE	0.07±0.03^ay^	0.06±0.04^ax^	/	0.13±0.01^ax^	/	/	/
**Ethers**								
13.26	Ethyl vinyl ether	1.21±1.08^ax^	0.59±0.16^ax^	1.95±0.49^ax^	1.30±0.89^bx^	1.11±0.83^bx^	3.20±1.72^ax^	/
**Other compounds**								
5.77	2-MF՛	2.43±2.47^ax^	2.01±1.64^ax^	/	2.64±1.88^ax^	1.84±1.02^ax^	/	/
5.97	Propionic anhydride	0.50±0.48^ax^	0.71±0.15^ax^	0.70±0.28^ax^	0.61±0.65^ax^	0.66±0.65^ax^	/	/
7.98	1,3-Propanediamine	0.41±0.25^bx^	0.34±0.23^bx^	0.88±0.15^ax^	1.31±1.42^ax^	0.39±0.22^ax^	/	M_9_
13.24	Diamide	1.58±1.28^ax^	0.93±0.66^ax^	/	1.69±0.95^ax^	0.94±0.61^ax^	/	/
14.11	1H-T-5-A	0.59±1.02^bx^	0.38±0.29^bx^	4.97±4.22^ax^	0.46±0.48^ax^	0.16 ±0.17^ax^	0.36±0.31^ax^	/
14.42	5-A-2-M-2H-T	0.82±1.34^ax^	0.22±0.16^ax^	0.54±0.32^ax^	0.43±0.42^ax^	0.23±0.16^ax^	0.26±0.17^ay^	/
14.69	2-Pentylfuran	0.64±0.19^bx^	3.13±2.44^ax^	/	/	0.81±0.66^ay^	/	5.8
17.77	2-MA	1.37±1.00^ax^	1.94±1.73^ax^	/	1.82±1.59^ax^	2.02±1.87^ax^	/	/

# RT ‒ Retention time; ST ‒ Sensory threshold. Each value was expressed as mean ± SD (n≥7) and kept three significant digits. ^a-b^Mean values in the different ages with different superscripts differ significantly (*P*<0.05) within the same muscle type; ^x-y^Mean values in the same ages with different superscripts differ significantly (*P* <0.05) within the different muscle type. DS՛ ‒ Dimethylsilanediol; 2,5-D-3-H ‒ 2,5-Dimethyl-3-hexanol; 2,4-D-3-H ‒ 2,4-Dimethyl-3-heptanone; 6-M-5-H-2-O ‒ 6-Methyl-5-hepten-2-one; S-(-)-1,2-E ‒ S-(-)-1,2-Epoxypropane; HC՛ ‒ Hexamethylcyclotrisiloxane; 1,3,5,7-C ‒ 1,3,5,7-Cyclooctatetraene; OCS՛ ‒ Octamethylcyclotetrasiloxane; 4,7-D ‒ 4,7-Dimethylundecane; DCS՛ ‒ Decamethylcyclopentasiloxane; DC՛ ‒ Dodecamethylcyclohexasiloxane; HC՛ ‒ Hexadecamethylcyclooctasiloxane; OC՛ ‒ Octadecamethylcyclononasiloxane; AAEE ‒ Acetic acid ethenyl ester; 2MF՛ ‒ 2-Methyltetrahydrofuran; 1H-T-5-A ‒ 1H-Tetrazol-5-amine; 5-A-2-M-2H-T ‒ 5-Amino-2-methyl-2H-tetrazole; 2-MA ‒ 2-Methylpentanoic anhydride. M_1_ ‒ 0.13-0.29 ng/g; M_2_ ‒ 400-1700 ng/g; M_3_ ‒ 25482.2 ng/g; M_4_ ‒ 810000-990000 ng/g; M_5_ ‒ 35400.2 ng/g; M_6_ ‒ 80-160 ng/g; M_7_ ‒ 41-82 ng/g; M_8_ ‒ 2205.25 ng/g; M_9_ ‒ 12400000 ng/g. Sensory threshold values in water were obtained from Gemert et al. (2011), Fan et al. (2018) and Shen et al., (2023).

**Figure 4:**
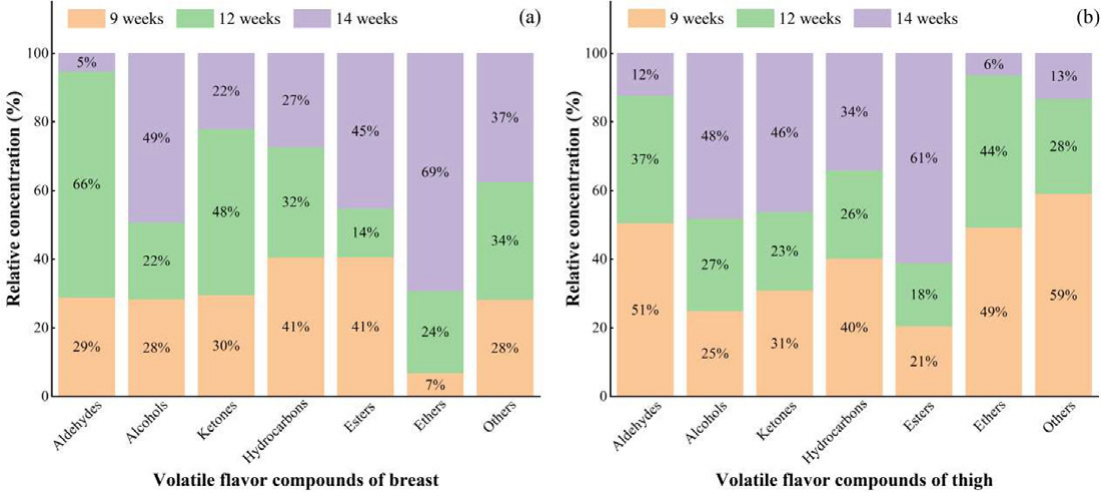
Percentage of volatile flavor substances in breast (A) and thigh (B) from XZ-FRCs at 9 to 14 wk.

As one of the major flavor components, the number of aldehydes (Zhang et al., 2021), whatever breast or thigh muscles, at 12 wk was far more than that at 9 and 14 wk (Table 3). The concentrations of seven flavor compounds from aldehydes, including heptanal, nonanal, decanal, dodecanal, tridecanal, tetradecanal, and pentadecanal, in breast muscles at 12 wk were significantly higher than those at 14 wk (*P* < 0.05) (Table 3). Besides, the proportions of aldehyde concentrations in breast and thigh muscles at 12 wk were also obviously higher than those at 14 wk (*P* < 0.05) (Figure 4). And with increasing age, the concentrations of pentanal, benzaldehyde and octanal in thigh muscles had an obvious decrease trend (*P* < 0.05). For other components, the number of alcohols, ketones, hydrocarbons, esters and other compounds at 12 wk was also more than that at 14 wk. The concentrations of most these compounds in breast or thigh muscles, such as 1-hexanol and trichloromethane, at 12 wk were obviously higher those at 14 wk, though several compounds like dimethylsilanediol, 3-heptanone, *etc*. had the opposite results (*P* < 0.05). The above phenomena suggested that the XZ-FRCs at 12 wk had a richer flavor than 14 wk.

In fact, many organic macromolecules, such as peptides and free amino acids, present in chicken are considered as the basic substrates on the formation of volatile flavor (Kosowska et al., 2017). In other words, the flavor characteristics in breast and thigh muscles from XZ-FRCs at different ages would depend on the types and contents of these macromolecules. The slaughter ages of XZ-FRCs might have some influence on the formation of these macromolecules, which would be necessary to study the relation between flavor compounds and these organic macromolecules in future work. In addition, the concentration of volatile compounds would also depend on their physicochemical characteristics (e.g., hydrophobicity, molecular weight) (Qi et al., 2021) and environmental factors in vivo (e.g., meat matrix, endogenous enzyme) (Qi et al., 2020; Deng et al., 2022). From Table 3, it was easy to find that the mean concentration of many compounds was close to the standard deviation of them, implying that these compounds had a weaker stability and a greater difference among individuals with same ages.

### Flavoring Nucleotides

As shown in Table 4, the slaughter ages at 9 to 14 wk had no significantly effect on hypoxanthine (HX) and inosine (I) contents in breast and thigh muscles from XZ-FRCs (*P* > 0.05). For XZ-FRCs at 14 wk, the contents of inosine 5ʹ-monophosphate (**5ʹ-IMP**), guanosine 5ʹ-monophosphate (**5ʹ-GMP**) and adenosine 5ʹ-monophosphate (**5ʹ-AMP**) in breast muscles were obviously higher than those in thigh muscles (*P* < 0.05). Compared with 14 wk, the thigh muscles at 12 wk had significantly higher 5ʹ-IMP and 5ʹ-GMP contents (*P* < 0.05), which were 2 main fresh nucleotides (Katemala et al., 2021; Sun et al., 2023) and had no changed obviously in breast muscles (*P* > 0.05). These results meant that the 12 wk as slaughter age of XZ-FRCs would have better flavor than 14 wk. Adversely, the content of 5ʹ-AMP in breast muscles at 14 wk was significantly higher than that at 9 to 12 wk (*P* < 0.05). However, the contents of 5ʹ-AMP and 5ʹ-GMP in breast and thigh muscles were no more than their corresponding sensory threshold (Table 4). Therefore, the 5ʹ-AMP has always been identified in free-range chicken muscles as a synergistic flavor substance (Yuan et al., 2021). The 5′-IMP content was much higher than its sensory threshold (25 mg/100g) and the other nucleotide contents (Table 4), which suggested that the 5′-IMP was the main flavor nucleotide of XZ-FRCs. The above changes of flavor nucleotides in XZ-FRCs at different ages may depend primarily on the activities of AMP deaminase (from AMP to IMP), 5′-nucleotidase (from IMP to I) and nucleoside phosphorylase (from I to Hx) (Hong et al., 2015; Lou et al., 2021).

**Table 4:** The contents of flavor nucleotides in breast and thigh muscles from XZ-FRCs at different ages (9–14 wk).^#^

Type	Age	Content (mg/100g wet sample)				
		5′-IMP	5′-GMP	5′-AMP	HX	I
Breast	9	183.70±18.77^ax^	4.00±0.42^ax^	6.32±1.39^bx^	14.43±4.94^ax^	46.98±13.97^ax^
	12	165.44±75.28^ax^	2.88±0.07^bx^	3.76±0.50^bx^	19.51±6.91^ax^	33.57±19.04^ax^
	14	199.56±17.15^ax^	3.09±0.30^bx^	11.17±1.48^ax^	18.91±2.23^ax^	58.65±28.48^ax^
Thigh	9	112.21±11.02^aby^	2.77±0.33^aby^	4.18±0.92^ax^	27.49±6.15^ay^	24.91±5.46^ax^
	12	173.00±67.03^ax^	3.44±0.35^ax^	6.88±3.94^ax^	17.27±6.51^ax^	36.76±14.40^ax^
	14	74.13±13.73^by^	2.05±0.05^by^	4.32±0.44^ay^	31.98±7.92^ax^	28.34±5.99^ax^
**Sensory threshold**		25	12.5	50	/	/

^a-c^Mean values in the different ages with different superscripts differ significantly (*P* < 0.05) within the same muscle type; ^x-y^Mean values in the same age with different superscripts differ significantly (*P* < 0.05) within the different muscle type; IMP, GMP, AMP, Hx and I were inosine 5ʹ-monophosphate, guanosine 5ʹ-monophosphate, adenosine 5ʹ-monophosphate, hypoxanthine and inosine, respectively; Taste threshold values of HX and I were not found in existing literatures; Taste threshold values of 5ʹ-IMP, 5ʹ-GMP and 5ʹ-AMP were obtained from Qi et al. (2017) and Yuan et al., 2021.

### Colligation Evaluation of Slaughter Age

**Figure 5:**
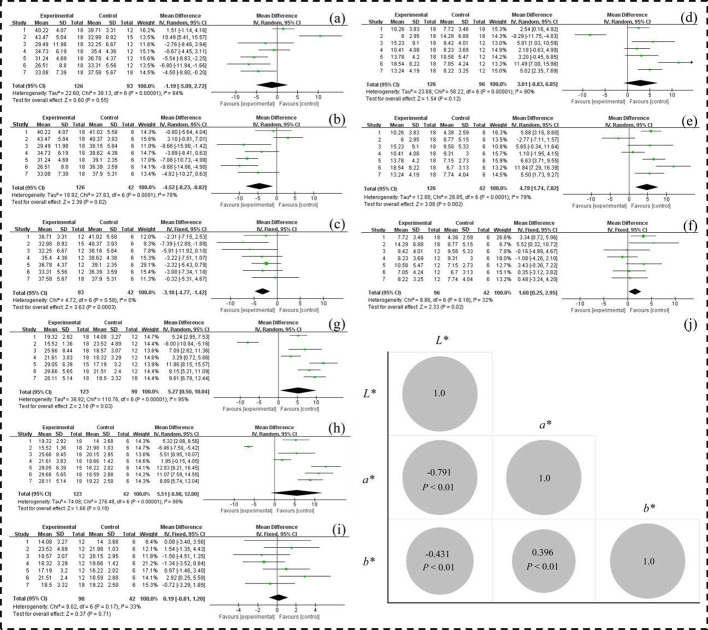
Results of meta-analysis (A–I) and Pearson correlation analysis (J) for CIE L*, a* and b* values. L* ‒ 9 wk vs. 12 wk (A), 9 wk vs. 14 wk (B), and 12 wk vs. 14 wk (C); a* ‒ 9 wk vs. 12 wk (D), 9 wk vs. 14 wk (E), 12 wk vs. 14 wk (F); b* 9 wk vs 12 wk (G), 9 wk vs. 14 wk (H), 12 wk vs. 14 wk (I).

The results of meta-analysis and Pearson correlation analysis for CIE L*, a* and b* values were shown in Figure 5. The L* and a* values of XZ-FRCs had no significance between 9 and 12 wk (*P* > 0.05), while they had significant difference not only between 9 and 14 wk, but also between 12 and 14 wk (*P* < 0.05) (Figures 5A–5F). For b* values of XZ-FRCs, the results of meta-analysis between 9, 12, and 14 wk were just the opposite with the L* and a* values (Figures 5G–5I). The changes of these parameters (CIE L*, a* and b*) did not have an obvious effect on the sensory evaluation of meat color in XZ-FRCs (data not shown). In other words, the change interval of above color parameters might be no more than the sensory threshold of human eye observation. Moreover, the L* values were highly negatively correlated with the a* values (Figure 5J), suggesting that the changes of L* values were caused largely by the a* values. Through the above analysis, the color parameters had not been applied in the final colligation score.

In general, the higher of shear force and centrifugal loss, the worse the meat quality in XZ-FRCs. Therefore, the weight coefficients of shear force and centrifugal loss were set to -1 in the calculation of colligation score. Nine flavor compounds as feature indexes were selected based on sensory thresholds (Table 2, Table 3, Table 4) and their weight coefficients were listed in Table 5. Finally, the result of colligation scores for XZ-FRCs was also shown in Table 5. The highest colligation score was observed in age of 12 wk, which was considered as a better slaughter age, compared with 9 and 14 wk. Furthermore, the weight of XZ-FRCs at 9 wk would increase significantly if feeding was continued. Therefore, it would be not a cost-effective selection when the slaughter age of XZ-FRCs was scheduled for 9 wk.

**Table 5:** Colligation scores of XZ-FRCs at different slaughter ages with 9 to 14 wk.

Index and colligation score	Weight coefficient	Score of XZ-FRCs at different ages		
		9 wk	12 wk	14 wk
**Feature indexes**				
Shear force	−1	−1.5	−1.97	−1.94
Centrifugal loss	−1	−1.42	−1.34	−2.0
Inosine 5ʹ-monophosphate	1	1.57	1.83	1.43
Glutamic acid	1	1.9	2.0	1.64
Histidine	−1	−1.97	−2.0	−1.84
Hexanal	1	1.42	1.74	0.62
1-Octen-3-ol	1	1.29	2.0	0.53
Heptanal	1	1.37	2.0	0.3
Octanal	1	1.39	1.8	0.3
Nonanal	1	1.33	1.84	0.53
Dodecanal	1	0	1.0	0
**Colligation score**				
	/	5.38	8.90	−0.43

## CONCLUSIONS

In this work, the effects of ages on meat qualities of XZ-FRCs and the relationship between them were studied to explore a better slaughter age. The results shown that the ages had significantly effect on color (CIE L*, a* and b* values), shear force, centrifugal loss and flavor of XZ-FRCs. Most of the quality characteristics in breast muscles from XZ-FRCs, whatever at same or different ages, were different with thigh muscles. The L* values were highly negatively correlated with a* values. Eleven feature indexes including shear force, centrifugal loss, inosine 5ʹ-monophosphate, glutamic acid, histidine, hexanal, 1-octen-3-ol, heptanal, octanal, nonanal and dodecanal were selected to explore a better slaughter age of XZ-FRC based on the calculation of colligation score. The highest score was obtained at 12 wk compared with 9 and 14 wk. Therefore, the XZ-FRC at 12 wk was considered as a better slaughter age, which would help the producers to improve the qualities of chicken meat.

## DISCLOSURES

The authors declare no conflicts of interest.
